# Prenatal Methylmercury, Postnatal Lead Exposure, and Evidence of Attention Deficit/Hyperactivity Disorder among Inuit Children in Arctic Québec

**DOI:** 10.1289/ehp.1204976

**Published:** 2012-09-21

**Authors:** Olivier Boucher, Sandra W. Jacobson, Pierrich Plusquellec, Éric Dewailly, Pierre Ayotte, Nadine Forget-Dubois, Joseph L. Jacobson, Gina Muckle

**Affiliations:** 1 Centre de Recherche du Centre hospitalier universitaire de Québec, Québec, Québec, Canada; 2 Department of Psychiatry and Behavioral Neurosciences, Wayne State University School of Medicine, Detroit, Michigan, USA; 3 Université de Sherbrooke, Sherbrooke, Québec, Canada; 4 Université Laval, Québec, Québec, Canada

**Keywords:** ADHD, attention, children, exposure, externalizing behavior, lead, mercury, methylmercury, polychlorinated biphenyls

## Abstract

Background: Prenatal exposure to methylmercury (MeHg) and polychlorinated biphenyls (PCBs) has been associated with impaired performance on attention tasks in previous studies, but the extent to which these cognitive deficits translate into behavioral problems in the classroom and attention deficit/hyperactivity disorder (ADHD) remains unknown. By contrast, lead (Pb) exposure in childhood has been associated with ADHD and disruptive behaviors in several studies.

Objectives: In this study we examined the relation of developmental exposure to MeHg, PCBs, and Pb to behavioral problems at school age in Inuit children exposed through their traditional diet.

Methods: In a prospective longitudinal study conducted in the Canadian Arctic, exposure to contaminants was measured at birth and at school age. An assessment of child behavior (*n* = 279; mean age = 11.3 years) was obtained from the child’s classroom teacher on the Teacher Report Form (TRF) from the Child Behavior Checklist, and the Disruptive Behavior Disorders Rating Scale (DBD).

Results: Cord blood mercury concentrations were associated with higher TRF symptom scores for attention problems and DBD scores consistent with ADHD. Current blood Pb concentrations were associated with higher TRF symptom scores for externalizing problems and with symptoms of ADHD (hyperactive-impulsive type) based on the DBD.

Conclusions: To our knowledge, this study is the first to identify an association between prenatal MeHg and ADHD symptomatology in childhood and the first to replicate previously reported associations between low-level childhood Pb exposure and ADHD in a population exposed to Pb primarily from dietary sources.

Mercury (Hg), polychlorinated biphenyls (PCBs), and lead (Pb) are widespread environmental contaminants known for their adverse effects on neurodevelopment (Grandjean and Landrigan 2006). Organic Hg, or methylmercury (MeHg), is the most neurotoxic form of Hg and is present in fish and marine mammals (Word Health Organization 1990). The adverse effects of acute MeHg exposure *in utero* have been documented following poisoning episodes that occurred in Japan (1953; 1964–65) and Iraq (1971–72) and include neurological symptoms and developmental delays (Amin-Zaki et al. 1974; Tsubaki and Irukayama 1977). In marine mammal- and fish-eating populations, chronic exposure to lower doses of MeHg during prenatal development has been associated with impairment in several domains of cognition including attention (Grandjean et al. 1997). However, the extent to which prenatal MeHg exposure is associated with behavioral problems at school age, in the absence of overt toxicological effects, remains unknown.

PCBs are synthetic organochlorine compounds (OCs) known for their long persistence in the environment. Like other OCs, their production and use has been banned or restricted in most industrialized countries, and population exposure to these chemicals now arises mainly from the ingestion of contaminated food (e.g., Moon et al. 2009). Several birth cohort studies have reported subtle cognitive alterations during childhood associated with prenatal PCB exposure, including response inhibition and attention deficits (Jacobson and Jacobson 2003; Stewart et al. 2005; reviewed by Boucher et al. 2009). In a recent prospective birth cohort study conducted near a PCB-contaminated harbor in New Bedford, Massachussets, higher PCB and OC levels in umbilical cord predicted attention deficit/hyperactivity disorder (ADHD)–like behaviors reported by classroom teachers (Sagiv et al. 2010).

Pb exposure has also been associated with impaired performance on neuropsychological tasks assessing attention and inhibition (Chiodo et al. 2004; Surkan et al. 2007). Prenatal Pb exposure is related to reduced IQ scores (Schnaas et al. 2006; Wasserman et al. 1998), and the relation between postnatal Pb exposure and ADHD symptoms and diagnoses is well established (e.g., Braun et al. 2006; Froehlich et al. 2009; Ha et al. 2009; Kim et al. 2010). Exposure to Pb during childhood has also been associated with conduct disorder (CD) (Braun et al. 2008), higher rates of criminal arrests in early adulthood (Wright et al. 2008), and teachers’ ratings of anxiety and social problems (Roy et al. 2009).

The Inuit population in Nunavik (Arctic Québec, Canada) is exposed to MeHg and PCBs through consumption of marine mammals and fish (Muckle et al. 2001), and is also exposed to Pb through the use of Pb pellets for game hunting (Lévesque et al. 2003). This study was designed to examine the relation of developmental exposure to MeHg, PCBs, and Pb to behavioral problems at 11 years of age as reported by the child’s classroom teacher.

## Methods

*Participants.* The participants were Inuit children from Nunavik, a region located north of the 55th parallel, about 1,500 km from Montréal. Most of the participants (*n* = 208) were initially recruited under the auspices of the Cord Blood Monitoring Program (1993–1998), which was designed to document prenatal exposure to a range of environmental contaminants and nutrients in newborns in Arctic Québec (Dallaire et al. 2001); the others (*n* = 57) were originally recruited for the Environmental Contaminants and Child Development Study (1996–2000; Muckle et al. 2001), and an additional 14 children had been involved in both studies. Mothers were contacted by phone, provided with information about the study protocol, and invited to participate with their children in the Nunavik Child Development Study. Inclusion criteria were age between 8.5 and 14.5 years, birth weight ≥ 2.5 kg, gestation duration ≥ 35 weeks, and no major birth defects, neurological or health problems, or pervasive development disorders. Written informed consent was provided by a parent of each participant, and oral assent was provided by each child. The research was approved by the Laval University and Wayne State University ethics committees and was conducted in accordance with ethical standards of the Helsinki Declaration (World Medical Association 2008).

Between September 2005 and February 2010, 294 children and their mothers participated in neurocognitive assessments in the three largest Nunavik villages. Participants who resided in other communities were transported by plane to one of the larger villages for testing. A maternal interview was conducted to provide information on demographic background and other factors including smoking and alcohol and drug use during pregnancy. Questionnaires for behavior assessment were filled by the child’s classroom teacher, who obtained the forms from our research nurse via the school principal. Questionnaires from eight participants were not returned by the classroom teacher, and two children were not assessed because they had not attended school since the beginning of the academic year. Of the 284 remaining children, 2 with a history of epilepsy, 1 with a history of head trauma requiring surgery, 1 with a history of meningitis associated with coma, and 1 with multiple sclerosis were excluded after data collection.

*Behavior assessments*. The Teacher Report Form (TRF) from the Child Behavior Checklist (Achenbach and Rescorla 2001) was completed by the child’s classroom teacher. The TRF contains 112 items, each of which is rated on a 3-point scale for applicability to the child: 0 = not true; 1 = somewhat or sometimes true; 2 = very true or often true. Eight syndrome scores are computed by summing the scores on specific items: Anxious/depressed, Withdrawn/depressed, Somatic complaints, Social problems, Thought problems, Aggressive, Rule-breaking behavior, and Attention problems. The first three syndrome scores are summed to compute the Internalizing problems score, and the Aggressive and Rule-breaking behavior scores are summed to obtain the Externalizing problems score. To limit the number of statistical comparisons, we restricted our analyses to Internalizing, Externalizing, and Attention problems. Because no normative data are available for Inuit children, raw scores for each individual scale were used in statistical analyses.

The Disruptive Behavior Disorders Rating Scale (DBD; Pelham et al. 1992) contains 45 behavioral descriptors based on the *Diagnostic and Statistical Manual of Mental Disorders, 4th edition* (DSM-IV; American Psychiatric Association 1994). It is designed to be completed by parents and teachers to provide information required for four clinical diagnoses: ADHD–Inattentive type, ADHD–Hyperactive-impulsive type, Oppositional Defiant Disorder (ODD), and Conduct Disorder (CD). Each behavioral descriptor is rated on a 4-point scale: 1 = never/rarely; 2 = sometimes; 3 = often; 4 = very often. Symptoms are considered to be present when rated either 3 or 4. A teacher report of at least six DSM-IV symptoms of ADHD–Inattentive type, six of ADHD–Hyperactive-impulsive type, four of ODD, and three of CD is necessary for the child to be characterized as presenting these disorders, based on the DSM-IV criteria. Because most (88%) children identified as CD were also identified as ODD, children meeting criteria for either of these diagnoses were grouped together in the statistical analyses.

*Biological samples.* Umbilical cord blood samples (30 mL) collected at birth for the Cord Blood Monitoring Program or Environmental Contaminants and Child Development Study were previously analyzed for concentrations of Hg, PCBs, Pb, polyunsaturated fatty acids, and selenium (Se) to indicate prenatal exposure. Child blood samples (20 mL) collected for the present study were analyzed for the same contaminants and nutrients to document current body burden. Contaminant and Se analyses were performed at the Centre de Toxicologie, Institut National de Santé Publique du Québec (Québec, Canada). Omega-3 fatty acid composition of plasma phospholipids was analyzed at the University of Guelph Lipid Analytical Laboratory (Guelph, Ontario, Canada). Detailed analytical procedures for cord and child blood samples are described in Supplemental Material, pp. 2–4 (http://dx.doi.org/10.1289/ehp.1204976).

*Confounding variables.* The following potential confounding variables were considered based on prior studies of the effects of environmental contaminants and our knowledge of socioeconomic and demographic factors associated with child development in the Inuit population: *a*) child characteristics: age, sex, birth weight, duration of gestation, adoption status (yes/no), and breast-feeding status (yes/no); *b*) maternal and family characteristics: age of biological mother at delivery, parity of biological mother before child’s birth, maternal education (years), marital status (single vs. married/living with partner), socioeconomic status (SES) based on the summation of predefined scores given for parental occupation status (lowest: unemployed/farm laborers/menial service workers; highest: higher executives, proprietors of large businesses, and major professionals) and education (lowest: < 7th grade; highest: graduate professionnal training; Hollingshead 1975), nonverbal reasoning ability (Raven Progressive Matrices; Raven et al. 1992), residential crowding (number of persons living in the house per room), and food insecurity (mother reporting at least 1 day without sufficient food or funds to purchase food in the month preceding the study; yes/no); *c*) seafood nutrients: docosahexaenoic acid (DHA) and Se concentrations in cord and child blood samples; and *d*) other prenatal exposures: maternal tobacco use (yes/no), binge drinking (at least one episode of ≥ 5 standard alcohol drinks; yes/no), and illicit drug use (yes/no) during pregnancy. When data on maternal substance use during pregnancy were available from previous assessments, the information obtained closest to delivery was used to optimize the accuracy of responses (1-month postpartum interview: *n* = 71; 5-year assessment: *n* = 83). Otherwise, information provided for the present study was used. Correlations between previous and current maternal reports of substance use during pregnancy were in the moderate-to-strong range (*r*_Φ_ between 0.42–0.80), suggesting reasonably robust validity of maternal reports provided a full decade after delivery.

*Statistical analyses.* Normality of distribution was inspected visually for each variable and checked for skewness (normality range: –2.0 to 2.0). The following variables, which exhibited log-normal distributions, were log transformed: cord and child blood Hg, PCB congener 153, and Pb; cord Se; parity; and each of the TRF outcome variables. A dichotomized (median split) variable was created for child Se because its distribution was neither normal nor log-normal. The following variables with extreme values (> 3 SDs from the mean) were recoded to one point greater than the highest observed non-outlying value, as recommended by Winer (1971) (number of outliers are indicated in parentheses): cord DHA (*n* = 2), child DHA (*n* = 1), child age (*n* = 7), duration of gestation (*n* = 1), residential crowding (*n* = 1), maternal education (*n* = 3), SES (*n* = 1), and Raven score (*n* = 1).

The relation of each of the exposure variables (cord and current concentrations of Hg, PCB-153, and Pb) to each TRF score was examined using multiple regression analysis. The relation between each of these exposures and each of the DBD-based diagnoses was examined using binary logistic regression analyses, in which the contaminant concentrations were divided into tertiles. Both sets of multivariate analyses included a set of potential confounding variables selected *a priori* based on previous research on the effects of neurotoxicants in school-age children (Froehlich et al. 2009): child age and sex (Pastor and Reuben 2008), SES (Froehlich et al. 2007), age of the biological mother at birth (Claycomb et al. 2004), maternal tobacco use during pregnancy, and birth weight (Nigg and Breslau 2007). Additionally, contaminant variables that predicted a given outcome at *p* < 0.20 were added to models of the effects of other contaminant exposures on the outcome to control for confounding without considerable loss in statistical power.

In a second set of analyses, covariates were selected using a forward strategy (Greenland and Rothman 1998). Specifically, potential confounders that correlated with the end point in question at *p* < 0.20 were added to models and retained if they altered the standardized regression coefficient for the contaminant by ≥ 10%, with order of entry determined by the strength of the correlation between the confounder and end point (starting with the variable showing the strongest correlation with the outcome; see Jacobson et al. 2008). Given recent criticisms against lipid standardization for PCB analysis (Schisterman et al. 2005), all analyses were also reconducted using cord and current plasma PCB values unadjusted for lipid values. Associations between contaminant variables and outcomes were considered significant when *p* ≤ 0.05 after control for confounders.

## Results

*Sample characteristics.* Descriptive statistics for the study sample are summarized in Table 1. About 25% of the mothers had given birth to their child before the age of 20 years, and < 20% had completed at least 11 years of schooling. About two of every five families reported food insecurity in the month preceding the study. Five children had current blood Pb concentrations above the threshold value of 10 μg/dL considered by U.S. and Canadian public health agencies to indicate risk for Pb neurotoxicity.

**Table 1:** Descriptive characteristics of the study sample.

Variables	*n*	Mean	Median	SD	Range	Percent
Child characteristics
Child age at assessment (years)	279	11.3	11.4	0.8	8.5–14.3
Child sex (% girls)	279	50.5
Birth weight (kg)	277	3.5	3.5	0.5	2.5–4.7
Duration of gestation (weeks)	279	39.1	39.0	1.5	35.0–44.0
Adoption status (% adopted)	279	16.5
Breast-feeding status (% yes)	272	74.6
Caregiver characteristics/family environment
Maternal age at delivery (years)	279	23.8	22.9	5.7	15.0–42.0
Parity before child’s birth	279	2.0	2.0	1.8	0.0–9.0
Marital status (% single)	279	27.3
Education (years of schooling)	278	8.5	9.0	2.5	0.0–16.0
Employment (% working)	277	71.8
SES scorea	279	28.6	28.0	11.8	8.0–66.0
Nonverbal reasoning abilityb	279	34.9	37.0	9.8	4.0–56.0
Language at interview (% primarily Inuktitut)	279	9.3
Residential crowding (no. of persons/room)	277	1.5	1.3	0.5	0.5–3.8
Food insecurity (% yes)c	277	38.3
Contaminants
Cord Hg (μg/L)	269	21.6	16.6	17.5	1.0–99.3
Current Hg (μg/L)	275	4.6	3.0	4.7	0.1–34.1
Cord PCB-153 (μg/kg fat)	268	123.1	93.6	100.5	9.7–653.6
Current PCB-153 (μg/kg fat)	274	73.7	45.9	83.2	3.5–809.5
Cord Pb (μg/dL)	269	4.7	3.7	3.3	0.8–20.9
Current Pb (μg/dL)	275	2.7	2.1	2.2	0.4–12.8
Seafood nutrients
Cord DHA (% phospholipids)	264	3.7	3.5	1.3	1.1–7.7
Current DHA (% phospholipids)	274	2.4	2.2	1.0	0.1–5.5
Cord Se (μg/L)	252	339.5	276.4	173.7	110.5–1579.2
Current Se (μg/L)	275	197.4	181.6	94.8	71.1–947.5
Other prenatal exposures
Tobacco smoke (% yes)	271	84.9
Binge drinking of alcohol (% yes)d	238	34.5
Illicit drug use (% yes)	240	30.0
aAssessed with the Hollingshead index, which is computed from predefined scores given for parental occupation status and education (Hollingshead 1975). bBased on the Raven Progressive Matrices (Raven et al. 1992). cFood insecurity was defined as mother reporting having not enough food to eat for her family at least 1 day in the preceding month. dBinge drinking corresponds to consumption of ≥ 5 standard drinks per occasion; 1 standard drink corresponds to 0.5 oz of absolute alcohol, which is equivalent to 350 mL of beer (12 oz), 175 mL of wine (6 oz), or 44 mL of liquor (1.5 oz).												

TRF behavior problem scores and the incidence of teacher ratings consistent with DSM-IV disruptive behavior disorder diagnoses according to the DBD are presented in Table 2. About 14% of the children were identified as displaying the behaviors that characterize ADHD–Inattentive type, and a similar proportion of children were identified as ADHD–Hyperactive-impulsive type. About one child of five was described by his or her teacher as exhibiting behaviors consistent with ODD and/or CD.

**Table 2:** Descriptive statistics for behavioral outcomes.

Variables	*n*	Mean	Median	SD	Range	Percent
Teacher report form (raw scores)
Internalizing problems	277	8.1	6.0	7.4	0.0–40.0
Externalizing problems	277	15.0	12.0	13.4	0.0–53.0
Attention problems	277	13.9	12.0	11.0	0.0–42.0
Teacher DBD (% meeting diagnostic criteria)
ADHD–Inattention type	279	14.3
ADHD–Hyperactivity type	279	12.9
ADHD–either type	279	21.5
ODD and/or conduct disorder	279	22.2

*Associations with child behavior problems on the TRF.* Before and after controlling for confounders, cord blood Hg concentrations were significantly associated with teacher-reported attention problems on the TRF (Table 3). Child blood Pb concentrations were significantly associated with teacher-reported externalizing problems. Cord and child blood PCB, cord Pb, and child Hg concentrations were not significantly associated with any of the behavior problem scores. Associations based on alternative regression models with confounders selected using a forward strategy were similar [see Supplemental Material, Table S1 (http://dx.doi.org/10.1289/ehp.1204976)]. Using cord and current plasma PCB values unadjusted for lipid values also did not alter the results (data not shown).

**Table 3:** Relation of contaminant exposures to TRF symptom scores (log transformed) [β-coefficient (95% CI)].

Contaminants (log)	Internalizing problems			Externalizing problems			Attention problems		
	Unadjusted	Adjusted	Unadjusted	Adjusted	Unadjusted	Adjusted
Cord blood												
Hg	0.09 (–0.03, 0.21)	0.09 (–0.04, 0.22)	0.08 (–0.04, 0.20)	0.05 (–0.07, 0.18)	0.13 (0.01, 0.25)	0.13 (0.00, 0.25)
PCB-153	–0.03 (–0.15, 0.09)	–0.04 (–0.17, 0.09)	–0.01 (–0.13, 0.11)	0.01 (–0.12, 0.13)	0.02 (–0.10, 0.14)	0.02 (–0.11, 0.16)
Pb	0.02 (–0.10, 0.14)	0.05 (–0.10, 0.19)	0.07 (–0.05, 0.19)	0.09 (–0.05, 0.23)	0.02 (–0.10, 0.14)	0.05 (–0.10, 0.19)
Current blood												
Hg	–0.02 (–0.14, 0.10)	–0.01 (–0.14, 0.11)	0.03 (–0.09, 0.15)	0.03 (–0.09, 0.16)	–0.04 (–0.15, 0.08)	–0.11 (–0.25, 0.03)
PCB-153	–0.02 (–0.14, 0.10)	–0.02 (–0.15, 0.10)	0.01 (–0.11, 0.13)	–0.04 (–0.16, 0.09)	–0.03 (–0.15, 0.09)	–0.11 (–0.24, 0.03)
Pb	0.03 (–0.03, 0.21)	0.06 (–0.07, 0.20)	0.17 (0.05, 0.29)	0.14 (0.01, 0.26)	0.16 (0.04, 0.27)	0.08 (–0.05, 0.21)
Values are standardized regression coefficients (β) and 95% confidence intervals (CIs) from linear regression analyses. Adjusted models include the following control variable: child age and sex, SES, age of the biological mother at birth, maternal tobacco use during pregnancy, and birth weight. Additionally, cord Hg was included in the regression models examining the associations between other contaminant variables and Attention problems, and child Pb was included in the models examining the associations between other contaminant variables and Externalizing problems and Attention problems because their correlations with these outcomes were at p < 0.20.												

*Associations with disruptive behavior disorders based on the DBD*. Results from logistic regression analyses relating cord Hg and child Pb to the DBD-based diagnoses are presented in Table 4. Compared with children in the lowest tertile of cord blood Hg concentration, children in the second and third tertiles were significantly more likely to be classified as having ADHD–Inattentive type based on the DBD, and children in the third tertile were also significantly more likely to be classified as having ADHD–Hyperactive-impulsive type. For current blood Pb concentrations, children in both the second and third tertiles were substantially more likely to be classified as ADHD–Hyperactive-impulsive type than children in the first tertile, though estimates were imprecise due to small numbers of cases in the lowest tertile of exposure [e.g., adjusted odds ratio (OR) = 5.52; 95% confidence interval (CI): 1.38, 22.12 for child Pb in the third vs. first tertile of exposure]. Similar results were obtained using alternative regression models in which confounders were determined using a forward selection approach, except for the association between prenatal Hg exposure (third tertile) and ADHD–Hyperactive-impulsive type, which was positive but not statistically significant [see Supplemental Material, Table S2 (http://dx.doi.org/10.1289/ehp.1204976)]. None of the other exposure variables significantly predicted any of the DBD-based diagnoses (data not shown).

**Table 4:** Relation of Hg and Pb exposures to DBD-based diagnoses.

Exposure	ADHD–Inattentive type			ADHD–Hyperactive-impulsive type			ODD and/or CD		
	No. of cases (%)	OR (95% CI)	AOR (95% CI)	No. of cases (%)	OR (95% CI)	AOR (95% CI)	No. of cases (%)	OR (95% CI)	AOR (95% CI)
Cord Hg (μg/L)											
1st tertile (1.0–11.2; n = 90)	6 (6.7)	(Referent)		7 (7.8)	(Referent)		17 (18.9)	(Referent)	
2nd tertile (11.4–22.7; n = 91)	16 (17.6)	2.99 (1.11, 8.02)	2.77 (1.00, 7.65)	8 (8.8)	1.14 (0.40, 3.30)	0.95 (0.30, 3.00)	21 (23.1)	1.29 (0.63, 2.64)	1.19 (0.56, 2.56)
3rd tertile (22.9–99.3; n = 88)	17 (19.3)	3.35 (1.25, 8.95)	2.87 (1.04, 7.94)	18 (20.5)	3.05 (1.20, 7.72)	2.92 (1.07, 8.04)	22 (25.0)	1.43 (0.70, 2.93)	1.39 (0.65, 2.98)
Child Pb (μg/dL)											
1st tertile (0.4–1.6; n = 90)	10 (11.1)	(Referent)		3 (3.3)	(Referent)		14 (15.6)	(Referent)	
2nd tertile (1.6–2.7; n = 94)	15 (16.0)	1.52 (0.64, 3.58)	1.06 (0.42, 2.66)	14 (14.9)	5.07 (1.40, 18.3)	4.01 (1.06, 15.23)	24 (25.5)	1.86 (0.89, 3.88)	1.90 (0.88, 4.11)
3rd tertile (2.7–12.8; n = 91)	15 (16.5)	1.58 (0.67, 3.73)	1.01 (0.38, 2.64)	18 (19.8)	7.15 (2.03, 25.2)	5.52 (1.38, 22.12)	23 (25.3)	1.84 (0.88, 3.85)	1.53 (0.67, 3.49)
Values are unadjusted odds ratio (OR), adjusted odds ratio (AOR), and 95% confidence intervals (CIs) from logistic regression analyses. Adjusted models include the following control variables: child age and sex, SES, age of the biological mother at birth, maternal tobacco use during pregnancy, and birth weight. Additionally, cord Hg was included in the regression models examining the association between child Pb and ADHD–Inattentive and ADHD–Hyperactive-impulsive types, and child Pb was included in the models examining the association between cord Hg and ADHD–Hyperactive-impulsive type and ODD/CD because their correlations with these outcomes were at p < 0.20.											

## Discussion

In this study we examined the relation of developmental exposure to MeHg, PCBs, and Pb to behavioral problems reported by teachers in school-age children. Prenatal exposure to MeHg was associated with greater attention problems and with a substantially increased risk of teacher-reported symptoms consistent with ADHD. Specifically, children with higher cord Hg concentrations were about four times more likely to be identified by their classroom teachers as exhibiting behaviors that characterize the inattentive type of ADHD, though estimates were somewhat imprecise. In contrast, postnatal Pb exposure was associated with greater externalizing problems and with symptoms consistent with the hyperactive-impulsive type of ADHD. PCB, prenatal Pb, and postnatal Hg exposures were not significantly associated with any of these behavioral outcomes, and none of these exposures were related to ODD and CD.

This is, to our knowledge, the first study to identify associations between prenatal MeHg exposure and behaviors that provide the DSM-IV diagnostic criteria for ADHD. The evidence of an increased risk of attention deficit reported here is consistent with findings from neuropsychological assessments of children in the Faroe Islands and findings from an event-related potential (ERP) study we conducted on a subsample of the children in the current study. In the Faroese, where the mean cord blood Hg concentration was similar to that found in our study, prenatal MeHg exposure was associated with impaired performance on attention tasks at 7 and 14 years of age (Debes et al. 2006; Grandjean et al. 1997). A recent reanalysis of those data suggested a specific effect on sustained attention (Julvez et al. 2010), a neuropsychological domain particularly affected in ADHD–Inattentive type (Egeland and Kovalik-Gran 2010). In the ERP study, we found evidence that prenatal MeHg exposure alters primary attentional mechanisms modulating early processing of sensory information (Boucher et al. 2010). It has been proposed that cortical disruption and alterations in neurotransmission, notably within the dopaminergic and GABAergic systems, are involved in the neurotoxicity of prenatal MeHg exposure (Newland et al. 2008). Because dopamine is believed to play a major role in the pathophysiology of ADHD (Del Campo et al. 2011), it is plausible that this mechanism is involved in the relation of MeHg to ADHD in children.

Associations with ADHD-type behaviors were observed at cord blood Hg concentrations > 11.4 μg/L. Although such exposure levels are common among children from Nunavik, relatively few children from the general Canadian and U.S. populations are exposed to such high Hg levels (e.g., Rhainds et al. 1999). However, the proportion of children exposed at these levels is likely to be higher among certain subgroups. For example, in a cohort initiated after 11 September 2001 in lower Manhattan, New York City, China-born Asians showed considerably higher cord blood Hg concentrations than non-Asians (mean, 17.0 vs. 3.73 μg/L for China-born Asians and non-Asians, respectively) (Lederman et al. 2008). The exposures in such subgroups are well within the range of exposures associated with attention and ADHD in the current study.

The results reported here contrast with those of a large birth cohort study conducted in the Seychelles Islands in which prenatal exposure was not associated with impaired performance on cognitive tasks or with an increased risk of ADHD-type behaviors (Myers et al. 2003). MeHg exposure arises mainly from fish consumption in the Seychelles study population, and failure to adjust statistically for seafood nutrients has been suggested as an explanation for the absence of any evidence of MeHg-related adverse effects within this cohort, since beneficial effects of seafood nutrients may counteract adverse effects of MeHg (Strain et al. 2008). In our study population, MeHg was associated with ADHD behaviors even without statistical adjustment for seafood nutrients, although MeHg and nutrient concentrations are moderately intercorrelated (Boucher et al. 2010). An alternative explanation to these diverging results points to different sources of exposure—marine mammal meat in the Inuit and Faroese—which is not eaten in the Seychelles. In addition to MeHg, marine mammals also contain an extensive array of contaminants (Letcher et al. 2010), some of which were measured in this study, which may contribute to and/or accentuate MeHg effects. Further studies are needed to resolve this issue.

Our results relating to postnatal Pb exposure replicate those of several previous studies where childhood Pb exposure was associated with ADHD (reviewed by Eubig et al. 2010). Studies that examined the relation of Pb exposure to inattention and hyperactivity separately have yielded inconsistent results, some showing stronger associations with inattentive symptoms (Chiodo et al. 2007; Kim et al. 2010; Roy et al. 2009) and others with hyperactivity and impulsivity (Nicolescu et al. 2010; Nigg et al. 2010). In the present study, Pb was associated with symptoms of the hyperactive-impulsive type, but not the inattentive type, of ADHD. Effects of Pb on hyperactive and impulsive behavior are well-documented in animal studies, as they represent core features of Pb neurotoxicity (Brockel and Cory-Slechta 1998; Moreira et al. 2001). Child blood concentrations were also associated with higher levels of impulsivity and irritability among Nunavik children at 5 years of age (Plusquellec et al. 2010). In an ERP assessment using a go/no-go paradigm conducted on a subsample from this cohort, postnatal Pb exposure was associated with higher rates of false alarms and with decreased brain activity (a smaller P3 component) in response to “no-go” trials (Boucher et al. 2012), suggesting a specific deficit in response inhibition that could be implicated in the association between Pb and ADHD. It has been proposed that the association of childhood Pb exposure with hyperactivity and impulsivity is mediated by effects of Pb on the development and function of the prefrontal cortex (Cecil et al. 2008; Trope et al. 2001). This brain area is involved in executive and impulsive control (Jurado and Rosselli 2007) and has been implicated in ADHD neuropathology (Faraone and Biederman 2002).

The association between Pb and ADHD–Hyperactive-impulsive type was observed at very low blood Pb concentrations—in children with blood Pb levels between 1.6 to 2.7 µg/dL. These results are consistent with recent evidence of adverse effects from postnatal Pb exposure at levels well below the 10 µg/dL risk level used by public health authorities (Canfield et al. 2003; Chiodo et al. 2004) and further confirm the need to redefine downward the tolerable level of exposure for children and to conduct interventions to reduce their exposure.

The absence of clear evidence of adverse effects of PCB exposure on child behavior in this study contrasts with a recent finding from the New Bedford (Massachusetts) cohort study, where prenatal exposure was associated with higher rates of teacher-reported ADHD-like behaviors (Sagiv et al. 2010). One factor that might account for the differences in results between these two studies relates to differences in the PCB mixtures that are found in each study area. Unlike the residents of New Bedford, the Nunavik Inuit live far from the PCB contamination sources. Consequently, they are more likely to be exposed to the more persistent, highly chlorinated PCB congeners that have accumulated in the marine food chain and reached the Arctic (Dewailly et al. 1993). By contrast, the children from New Bedford were exposed to higher concentrations of the lower chlorinated, mono-*ortho*, and dioxin-like PCB congeners (Korrick et al. 2000), which may be attributable to the specific PCB mixtures that were used in New Bedford area industries, and to dechlorination processes that have altered PCB congeners in the New Bedford ecosystem (Brown and Wagner 1990; Burse et al. 1994).

The strengths of this study include our ability to control for confounding by other contaminants present in seafood—specifically for confounding of the association between cord Hg and outcomes by child Pb, and for confounding of the association between child Pb and outcomes by cord Hg. Another innovative aspect of this study is that we assessed prenatal and childhood intake of nutrients and evaluated them as potential confounders in the neurotoxicant–behavior relationships. Among the limitations of this study is that the maternal report of substance use during pregnancy was obtained in many cases about a decade after delivery. We tried to minimize this limitation by using data obtained at 1 month or 5 years postpartum when available, although this was not possible for the entire sample. Another limitation is that we do not have formal diagnoses for ADHD, but rather ADHD classifications based on behavior ratings provided by the classroom teachers. Although the elementary school classroom is an optimal context for observing the behaviors that characterize ADHD, and teachers are in a unique position to compare these behaviors across children, clinical diagnoses of ADHD typically also incorporate observations from other sources, particularly parents. Finally, we were not able to control for family history of ADHD. Because ADHD was rarely recognized in the previous generation in Nunavik, this potential confounding factor could not be reliably assessed.

## Conclusions

To our knowledge, this study is the first to report an association between prenatal MeHg exposure and ADHD symptomatology at school age. The associations with teacher-reported ADHD symptoms observed in the current study suggest that adverse effects of prenatal MeHg on attention previously reported based on neuropsychological assessments may be clinically significant, and may interfere with learning and performance in the classroom. This study also suggests that prenatal MeHg exposure may be a risk factor for attention problems in diverse ethnic groups from Southern Canada and the United States who may be exposed to similar levels of MeHg through their diet. Although the main source of Pb exposure in our study population—lead shot (as revealed by blood Pb isotope ratios; Lévesque et al. 2003)—is unique in the Pb exposure literature, this study replicates previous findings linking low-level childhood Pb exposure to ADHD. Our results support the need for local interventions intended to reduce prenatal exposure to MeHg and childhood exposure to Pb. Additionally, because MeHg exposure in the Arctic is attributable primarily to long-range transport of Hg from developing countries, international actions and conventions aimed at limiting Hg emissions are urgently needed.

## Supplemental Material

(147 KB) PDF
