# Supplementary material for: Prenatal Methylmercury, Postnatal Lead Exposure, and Evidence of Attention Deficit/Hyperactivity Disorder among Inuit Children in Arctic Québec
Source: Environ Health Perspect. 2012 Sep 21;120(10):1456–61. doi: 10.1289/ehp.1204976 (PMC3491943; doi:10.1289/ehp.1204976)
Supplement: (147 KB) PDF [file ehp.1204976.s001.pdf]

Supplemental Material

Prenatal Methylmercury, Postnatal Lead Exposure, and Evidence of Attention Deficit Hyperactivity Disorder among Inuit Children in Arctic Québec

Olivier Boucher[1], Sandra W. Jacobson[2], Pierrich Plusquellec[1,3], Éric Dewailly[1,4], Pierre Ayotte[1,4], Nadine Forget-Dubois[1,4], Joseph L. Jacobson[2], Gina Muckle[1,4]

[1]Centre de Recherche du CHUQ, Québec, Québec, Canada.

[2]Department of Psychiatry and Behavioral Neurosciences, Wayne State University School of Medicine, Detroit, Michigan, USA.

[3]Université de Sherbrooke, Sherbrooke, Québec, Canada.

[4]Université Laval, Québec, Québec, Canada.

Corresponding author:
Gina Muckle, PhD.
Centre de recherche du CHUQ
Édifice Delta 2, Bureau 600
2875, boulevard Laurier, 6e étage
Québec (Qc), Canada G1V 2M2.
Tel: 1 418 656 4141, ext. 46199.
Fax: 1 418 654 2726.
E-mail: gina.muckle@psy.ulaval.ca

Analytical procedures for contaminant and nutrient analyses

Total Hg concentrations in umbilical cord blood samples were determined using cold vapour atomic absorption spectrometry (Pharmacia Model 120; Pharmacia, Piscataway, NJ, USA). Cord blood Pb levels were determined by graphite furnace atomic absorption with Zeeman background correction (Perkin Elmer model ZL 4100; Perkin Elmer, Norwalk, CT, USA). PCB congeners were measured in purified cord and child plasma extracts using gas chromatography (HP 5890 Series II Plus) equipped with a 30-m long DB-5 capillary column (J&W Scientific, Folsom, CA, USA) and HP 5890B mass spectrometer (Agilent) according to the method described by Dallaire et al. (2009). The 14 most prevalent PCB congeners [International Union of Pure and Applied Chemistry (IUPAC) nos. 28, 52, 99, 101, 105, 118, 128, 138, 153, 156, 170, 180, 183, 187) were measured in purified cord plasma extracts using high-resolution gas chromatography (Hewlett-Packard HP5890A; Hewlett-Packard, Palo Alto, CA, USA), with two capillary columns (Hewlett-Packard Ultra I and Ultra II) and dual Ni-63 electron capture detectors. For the present study, PCB congener 153, expressed on a lipid basis, was used as an indicator of total PCB exposure. Cord Se concentrations were determined by inductively coupled plasma mass spectrometry (ICP-MS) on a Perkin Elmer Sciex Elan 6000 instrument.

Total Hg, Pb, and Se concentrations in child blood samples were determined by ICP-MS (Perkin Elmer Sciex Elan 6000 ICP-MS instrument for Pb and Se; PE DRC II instrument for Hg). The limits of detection for cord sample analyses were 0.2 µg/L for blood Hg and Pb, 0.1 µmol/L for blood Se, and 0.02 µg/L for all PCB congeners in plasma. Limits of detection for child blood sample analyses were 0.1 µg/L for Hg, 0.002 µg/dL for Pb, 0.09 µmol/L for Se, and were less than 0.05 µg/L for all PCB congeners except for PCB-52 (0.15 µg/L). A value equal to

half the limit of detection of the analytical method was entered in the database whenever a substance was not detected (child Hg: $n = 1$; other substances were detected in all samples).

Total phospholipids were isolated from the lipid extract by thin-layer chromatography using heptane:isopropyl ether:acetic acid (60:40:3, v/v/v) as the developing solvent. After transmethylation using BF3/methanol, the fatty acid profile was determined by capillary gas-liquid chromatography. Concentrations of docosahexaenoic acid (DHA) in cord and at time of testing were expressed as percentages of the total area of all fatty acid peaks from C14:0 to C24:1 (percent weight).

References

Dallaire R, Dewailly E, Pereg D, Dery S, Ayotte P. 2009. Thyroid function and plasma concentrations of polyhalogenated compounds in Inuit adults. Environ Health Perspect 117:1380-1386.

Table S1. Results from linear regression analyses for the relation of contaminants to TRF symptom scores using a forward strategy for covariate selection.

	Internalizing problems	Externalizing problems	Attention problems
Contaminants (log)	β-coefficient (95% CI)	β-coefficient (95% CI)	β-coefficient (95% CI)
Cord blood			
Hg	0.10 (-0.02, 0.22) [a,b,c]	0.03 (-0.10, 0.17) [a,c,e,f,g,h,i,j]	0.12 (0.00, 0.25) [a,f,g,l,n]
PCB 153	-0.06 (-0.19, 0.07) [a,b,c,d]	-0.02 (-0.15, 0.11) [a,c,e,f,g,h,j]	0.05 (-0.09, 0.19) [a,b,c,d,f,g,l,n,o]
Pb	-0.05 (-0.17, 0.08) [a,c,d]	0.03 (-0.10, 0.17) [a,f,g,h,i,j,k,l]	0.06 (-0.08, 0.19) [a,d,l,m,n]
Current blood			
Hg	0.06 (-0.09, 0.20) [a,b,c,e,f]	0.09 (-0.05, 0.22) [a,c,e,f,g,k,m]	0.02 (-0.13, 0.16) [a,b,c,e,f,g,l,m,n]
PCB 153	-0.06 (-0.20, 0.08) [a,b,c,d,e,f]	0.01 (-0.12, 0.14) [a,c,e,f,g,h,i,k,m]	-0.07 (-0.22, 0.08) [a,b,c,d,e,f,g,l,m,n]
Pb	0.07 (-0.05, 0.19) [a,c,d]	0.13 (0.01, 0.24) [a,c,k]	0.10 (-0.03, 0.22) [a,c,d,f,l,n]

Abbreviations: Hg, mercury; Pb, lead; PCB 153, polychlorinated biphenyl congener 153.

Values are standardized regression coefficients (β) and 95% confidence interval (CI) from a multivariate model including the selected control variables. Superscripts indicate control variables included in the final model for each of the regression analyses: [a]maternal Raven; [b]child DHA; [c]child Se; [d]cord Hg; [e]child Pb; [f]breastfeeding; [g]maternal smoking during pregnancy; [h]adoption status; [i]residential crowding; [j]maternal binge drinking during pregnancy; [k]food security; [l]child gender; [m]maternal education; [n]maternal age; [o]parity.

Table S2. Results from logistic regression analyses for the relation of Hg and Pb exposures to DBD-based diagnoses using a forward strategy for covariate selection.

	ADHD – Inattentive type		ADHD – Hyperactive-impulsive type		ODD and/or CD	
Exposure	AOR	(95% CI)	AOR	(95% CI)	AOR	(95% CI)
Cord Hg (µg/L)						
1st tertile (1.0 - 11.2)	(referent)		(referent)		(referent)	
2nd tertile (11.4 - 22.7)	3.49	(1.17, 10.47)	0.81	(0.27, 2.48)	1.48	(0.71, 3.10)
3rd tertile (22.9 - 99.3)	4.63	(1.54, 10.94)	2.06	(0.73, 5.82)	1.48	(0.71, 3.08)
Child Pb (µg/dL)						
1st tertile (0.4 - 1.6)	(referent)		(referent)		(referent)	
2nd tertile (1.6 - 2.7)	1.45	(0.59, 3.55)	4.89	(1.35, 17.71)	1.82	(0.87, 3.84)
3rd tertile (2.7 - 12.8)	1.11	(0.41, 3.04)	4.56	(1.19, 17.49)	1.77	(0.79, 3.93)

Abbreviations: ADHD, Attention deficit hyperactivity disorder; CD, Conduct disorder ; DBD, Disruptive Behavior Disorder Rating Scale; Hg, mercury; ODD, Oppositional deficant disorder; Pb, lead. Values are adjusted odds ratio (AOR) and 95 % confidence interval (CI). Multivariate models for ADHD – inattentive type include child gender, residential crowding, and child PCB 153. Multivariate model relating cord Hg to ADHD – hyperactive-impulsive type includes child Pb and cord Se; model relating child Pb to ADHD – hyperactive-impulsive type includes child gender and residential crowding. Multivariate models for ODD/CD include child Se, and the model relating child Pb to ODD/CD also includes child gender (Pb).
